# HIV-1 induces cytoskeletal alterations and Rac1 activation during monocyte-blood–brain barrier interactions: modulatory role of CCR5

**DOI:** 10.1186/1742-4690-11-20

**Published:** 2014-02-26

**Authors:** Shawna M Woollard, Hong Li, Sangya Singh, Fang Yu, Georgette D Kanmogne

**Affiliations:** 1 Department of Pharmacology and Experimental Neuroscience, University of Nebraska Medical Center, Omaha, Nebraska 68198-5800, USA; 2 Department of Biostatistics, University of Nebraska Medical Center, Omaha, Nebraska 68198-4375, USA

**Keywords:** HIV-1, Human brain endothelial cells, Monocytes, Macrophages, Cytoskeletal proteins, Maraviroc, TAK-779

## Abstract

**Background:**

Most HIV strains that enter the brain are macrophage-tropic and use the CCR5 receptor to bind and infect target cells. Because the cytoskeleton is a network of protein filaments involved in cellular movement and migration, we investigated whether CCR5 and the cytoskeleton are involved in endothelial-mononuclear phagocytes interactions, adhesion, and HIV-1 infection.

**Results:**

Using a cytoskeleton phospho-antibody microarray, we showed that after co-culture with human brain microvascular endothelial cells (HBMEC), HIV-1 infected monocytes increased expression and activation of cytoskeleton-associated proteins, including Rac1/cdc42 and cortactin, compared to non-infected monocytes co-cultured with HBMEC. Analysis of brain tissues from HIV-1-infected patients validated these findings, and showed transcriptional upregulation of Rac1 and cortactin, as well as increased activation of Rac1 in brain tissues of HIV-1-infected humans, compared to seronegative individuals and subjects with HIV-1-encephalitis. Confocal imaging showed that brain cells expressing phosphorylated Rac1 were mostly macrophages and blood vessels. CCR5 antagonists TAK-799 and maraviroc prevented HIV-induced upregulation and phosphorylation of cytoskeleton-associated proteins, prevented HIV-1 infection of macrophages, and diminished viral-induced adhesion of monocytes to HBMEC. Ingenuity pathway analysis suggests that during monocyte-endothelial interactions, HIV-1 alters protein expression and phosphorylation associated with integrin signaling, cellular morphology and cell movement, cellular assembly and organization, and post-translational modifications in monocytes. CCR5 antagonists prevented these HIV-1-induced alterations.

**Conclusions:**

HIV-1 activates cytoskeletal proteins during monocyte-endothelial interactions and increase transcription and activation of Rac1 in brain tissues. In addition to preventing macrophage infection, CCR5 antagonists could diminish viral-induced alteration and phosphorylation of cytoskeletal proteins, monocyte adhesion to the brain endothelium and viral entry into the central nervous system.

## Background

Despite the successes of antiretroviral therapy, the brain remains a major target for HIV infection and a major viral reservoir. This viral infection of the CNS commonly results in behavioral, motor, and cognitive impairments termed HIV-1-associated neurocognitive disorders (HAND) [1,2]. Disease pathology is characterized by the presence of microglial nodules and multinucleated giant cells, and includes brain atrophy, gliosis, and neuronal loss, all collectively termed HIV encephalitis (HIVE) [3]. HIV-infection of the CNS is fueled by viral infection and immune activation of mononuclear phagocytes (MPs), which promote monocyte trafficking across the blood–brain barrier (BBB) and spread of infection to resident brain cells [4].

HIV enters target cells by binding the envelope glycoprotein gp160 to the CD4 receptor and/or co-receptors such as CCR5 and CXCR4 [5-8]. The new class of antiretroviral drugs called entry inhibitors act by binding the CD4 receptor, CCR5 or CXCR4 co-receptors, to prevent viral binding and entry into cells. One of those entry inhibitors, maraviroc, is a small molecule CCR5 antagonist that is currently FDA-approved for the treatment of patients infected with macrophage (M)-tropic HIV strains [9-12]. TAK-779 is another small molecule non-peptide CCR5 antagonist that has been shown to suppress HIV-1 envelope-mediated membrane fusion [13] and reduce inflammation [14]. We previously demonstrated that human brain microvascular endothelial cells (HBMEC) lack the CD4 receptor but expressed both CCR5 and CXCR4 co-receptors [15].

Most HIV strains that cross the BBB, enter the brain, and infect CNS cells are M-tropic and use CCR5 to enter and infect target cells [16,17]. Therefore, we hypothesized that CCR5 plays a major role in monocyte-brain endothelium interactions and HIV entry into the CNS and that CCR5 blockers would abrogate these effects. Because the cytoskeleton is responsible for cellular morphology and motility, we further hypothesized that HIV-induced monocyte-endothelial interactions and trans-endothelial migration involve cytoskeletal changes and that CCR5 blockers would also affect these changes. In the current study, we used a cytoskeleton phospho-antibody array to investigate changes in the expression and activation of cytoskeleton-associated proteins in monocytes following HIV-1 infection and endothelial interaction. We further used CCR5 antagonists (TAK-779 and maraviroc) and antibodies to determine the role of CCR5 on HIV-1 infection of monocytes-derived macrophages (MDM), monocyte-endothelial interaction, and cytoskeletal changes. Data suggest that interaction of HIV-infected monocytes with HBMEC is associated with altered expression and activation of monocytes cytoskeletal proteins. CCR5 blockers can reverse those changes and prevent MDM infection. Most importantly in support of these findings, we showed increased transcription of cortactin gene (*CTTN*) and *RAC1*, and increased phosphorylation of Rac1 at serine-71 (S71) in brain tissues of HIV-1-infected patients. Confocal imaging showed that phospho-Rac1 was mostly expressed in brain macrophages and blood vessels. Cortactin and Rac1 are cytoskeletal proteins that have been shown to be involved in cytoskeletal remodeling, cellular motility, cell-cell adhesion, and leukocyte transmigration [18-20]. This suggests that HIV-1-induced endothelial-monocyte interactions and transmigration of infected MPs into the brain is associated with increased transcription, expression and activation of MPs cytoskeletal proteins, and CCR5 blockers could diminish these alterations.

## Results

### CCR5 blockers prevent HIV-1 infection of macrophages

**Figure 1:**
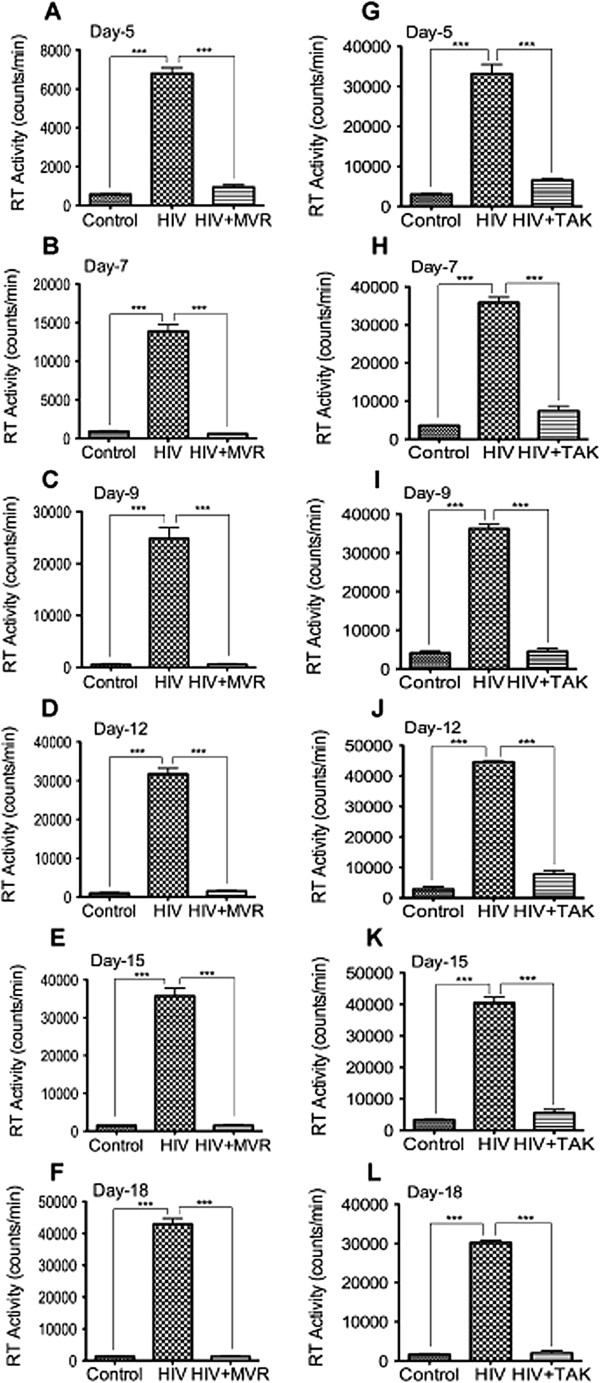
**CCR5 antagonists inhibits HIV-1 infection in MDM.** MDM were infected with the macrophage tropic HIV-1_ADA_, with or without maraviroc or TAK-779 (5 μM) in the media. Controls consisted of mock-infected cells. Culture supernatants were then collected every 2 or 3 days from day-5 to day-18 post-infection (p.i) and viral replication estimated by quantifying reverse transcriptase activities. From day-5 to day-18 p.i., both maraviroc **(A-F)** and TAK-779 **(G-L)** inhibited HIV-1 infection of MDM (***P < 0.001). This is representative data from 3 independent experiments with 3 different human donors, with each donor tested in triplicate.

CCR5 blockers such as maraviroc have been shown to block entry of M-tropic HIV into target cells [9], and maraviroc is currently FDA-approved for the treatment of individuals infected with M-or dual-tropic HIV [9-12]. To confirm the effects of CCR5 blockers *in vitro*, we infected human MDM with HIV-1 in the presence of TAK-779 or maraviroc and assessed viral replication by reverse transcriptase assay. Both maraviroc (Figure 1A-F) and TAK-779 (Figure 1G-L) significantly diminished MDM infection. From day-5 to day-18 post-infection (p.i), maraviroc diminished MDM infection by 7.2- to 44-fold (Figure 1A-F), while TAK-779 diminished MDM infection by 4.8- to 15.3-fold (Figure 1G-L). Additional experiments showed that TAK-779 and maraviroc concentrations as low as 0.05 μM significantly decreased viral infection (data not shown).

### Effects of CCR5 blockers on cellular viability and brain endothelial barrier functions

**Figure 2:**
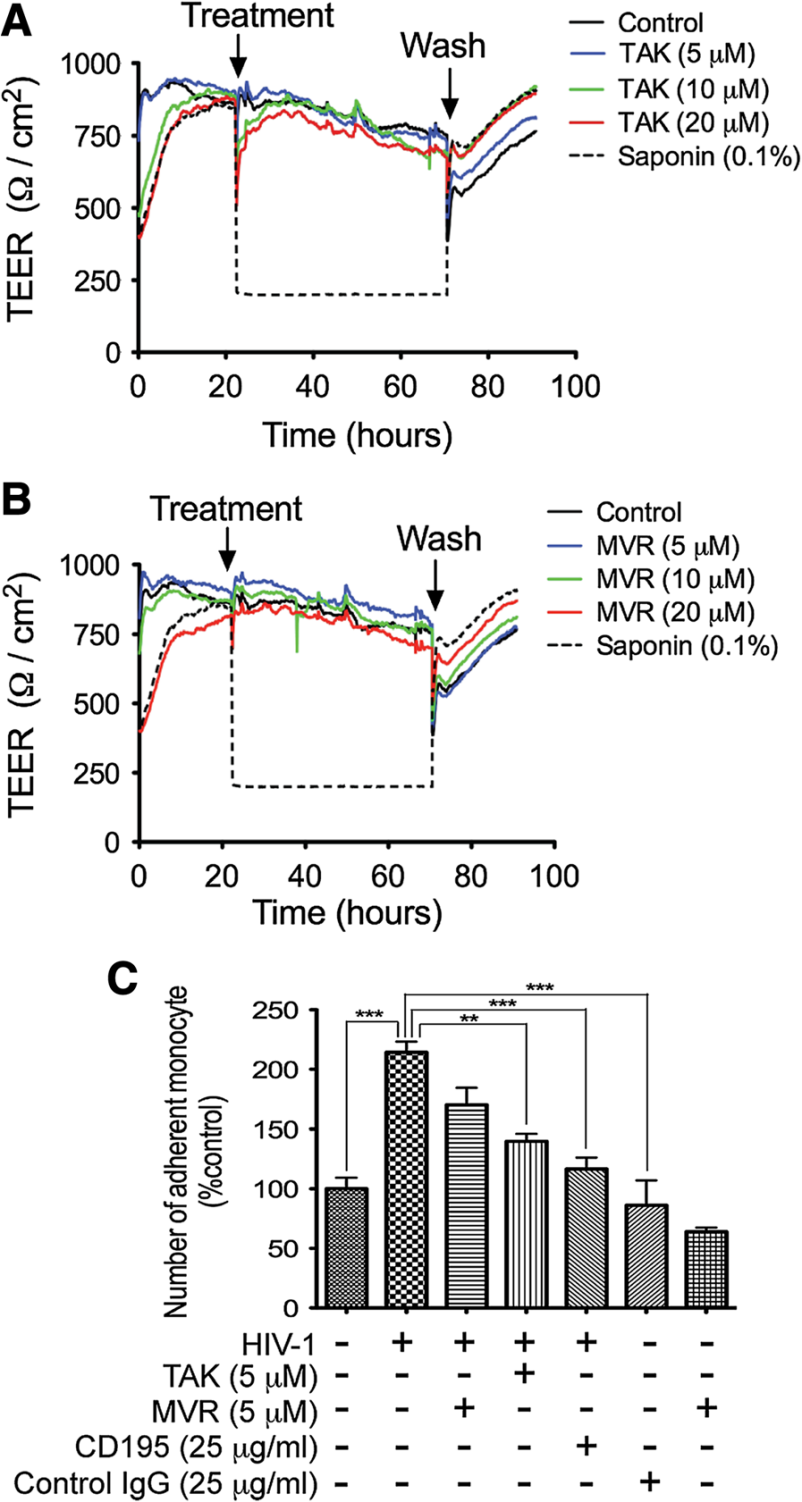
**Effects of CCR5 antagonists on the BBB integrity and function.** Confluent HBMEC monolayers were treated with TAK-799 **(A)** or maraviroc (MVR) **(B)**, and TEER measured in real-time before and after exposures. At 5, 10, or 20 μM, TAK-799 **(A)** or maraviroc **(B)** did not alter TEER. TEER values were similar during treatment and when cells were washed to remove CCR5 antagonists. Controls consisted of mock-treated cells and cells treated with 0.1% saponin. Saponin-induced decrease in TEER was reversed upon removal of saponin from the media. **(C)** HIV-1 increased monocyte adhesion to HBMEC and CCR5 antagonists and neutralizing CCR5 antibodies decreased viral-induced monocyte adhesion (**P < 0.01; ***P < 0.001).

To ensure that the observed anti-viral effects of CCR5 blockers were not due to inadvertent drug toxicity on macrophages, we tested the effects of TAK-779 and maraviroc on MDM viability. Results from day-5 to day-12 p.i. showed that TAK-779 or maraviroc did not alter MDM viability (data not shown). To determine whether CCR5 blockers could alter endothelial barrier properties and function, we assessed their effects on the brain trans-endothelial electrical resistance (TEER). Exposure of HBMEC to 5, 10, or 20 μM TAK-779 (Figure 2A) or maraviroc (Figure 2B) did not alter TEER. HIV-1 infection increased monocyte adhesion to HBMEC, and both CCR5 antagonists and neutralizing CCR5 antibodies significantly decreased HIV-induced monocyte adhesion (Figure 2C).

### Increased expression of cytoskeletal proteins in HIV-infected monocytes following monocyte-endothelial interactions

During leukocytes interactions with other immune cells, cellular cytoskeletons undergo major changes and reorganization that facilitates leukocyte motility and migration [21-23]. We determined the cytoskeleton-associated protein changes in monocytes during monocyte-endothelial communication in the presence or absence of the CCR5 blocker TAK-779. Protein microarray showed that compared to non-infected monocytes, two-hour co-culture of HIV-infected monocytes with HBMEC induced upregulation of cytoskeleton-associated proteins in monocytes, with 13 proteins upregulated by 2-fold or more (Table 1), while expression of the remaining 128 cytoskeleton-associated proteins was not significantly changed or did not meet the 2-fold cut-off. TAK-779 prevented HIV-induced upregulation of cytoskeleton-associated proteins during monocyte-endothelial interactions (Table 1, Additional file 1: Figure S1).

**Table 1:** Differently expressed total proteins in HIV-infected-monocytes co-cultured with HBMEC

**Protein**	**HIV vs. control fold change**	**P-value**	**HIV vs. TAK fold change**	**P-value**	**Location**
Src	3.05	5.09E-10	0.67	3.34E-05	Cytoplasm
MKK3/MAP2K3	2.78	3.62E-09	0.41	1.16E-11	Cytoplasm
PKC alpha	2.56	7.34E-08	0.57	6.81E-06	Cytoplasm
p130Cas	2.51	2.06E-08	0.54	1.16E-11	Plasma membrane
MKK7/MAP2K7	2.41	5.47E-09	0.45	1.16E-11	Cytoplasm
CaMK2-beta/gamma/delta	2.40	1.61E-09	0.69	3.61E-04	Nucleus
Ezrin	2.22	4.15E-10	0.50	1.16E-11	Plasma membrane
PLC beta3	2.17	3.62E-09	0.48	1.09E-06	Cytoplasm
c-Raf	2.07	1.99E-11	0.57	1.29E-11	Cytoplasm
PLC-beta	2.04	7.50E-07	0.53	2.09E-10	Cytoplasm
WAVE1	2.02	3.19E-06	1.17	1.61E-03	Nucleus
LIMK1	1.99	1.99E-11	0.46	3.08E-11	Cytoplasm
Myosin regulatory light chain 2	1.99	5.47E-09	0.63	7.03E-11	Cytoplasm

HBMEC: human brain microvascular endothelial cells; HIV: human immunodeficiency virus-1.

Unadjusted P values, P values adjusted for multiple comparisons using Tukey post-hoc test, and P values adjusted for false discovery rate using the Benjamini & Hochberg method were all significant. Table show P values adjusted using the Benjamini & Hochberg method.

Src: v-src sarcoma viral oncogene homolog; MKK3/MAP2K3: mitogen-activated protein kinase kinase3; PKC: protein kinase C; p130Cas: breast cancer anti-estrogen resistance 1; MKK7/MAP2K7: mitogen-activated protein kinase kinase 7; CAMK2: calcium/calmodulin-dependent protein kinase-2; PLC: phospholipase C; c-raf: v-raf-1 murine leukemia viral oncogene homolog 1; WAVE1: WAS protein family 1; LIMK1: LIM domain kinase 1.

### Altered phosphorylation of cytoskeleton-associated proteins in HIV-infected monocytes during monocyte-endothelial interactions

Post-translational modifications such as phosphorylation play a major role in the regulation of protein function, protein-protein interactions, and cellular function [22]. Because minor changes in phosphorylation level can have functional significance, we used a 1.5-fold change cut-off to analyze proteins differentially phosphorylated in HIV-1-infected monocytes following monocyte-endothelial co-culture, compared to infected monocytes treated with the CCR5 blocker and non-infected monocytes co-cultured with HBMEC. Normalization of each phospho-protein to the expression of its corresponding total protein showed that phosphorylation of 9 proteins increased by 1.5-fold or more, while phosphorylation of 12 proteins decreased by 1.5-fold or more, and 33 proteins had no significant change in phosphorylation level. Normalization of protein expression to the sample’s actin levels revealed increased phosphorylation in 33 proteins, decreased phosphorylation in 7 proteins, while 25 proteins had no significant change in phosphorylation levels. Phospho-proteins that showed significant differential expression using both normalization methods included 3 proteins with decreased phosphorylation and 8 cytoskeleton-associated proteins with significantly increased phosphorylation (Table 2). HIV-1 infection and endothelial-monocyte interactions increased the phosphorylation of Merlin (Ser518), vasodilator-stimulated phosphoprotein (VASP) (Ser157), Rac1(S71), cortactin (Tyr421), and ERK1/2(Tyr204/202) by 4.5 to 6.3-fold, 4-fold, 2.3- to 3.6-fold, 2 to 3-fold, and 2.4 to 2.6-fold respectively (Table 2). TAK-779 prevented HIV-induced phosphorylation of these cytoskeleton-associated proteins during monocyte-endothelial interactions (Table 2, Additional file 1: Figure S1). Ingenuity pathway analysis (IPA) of differentially expressed and phosphorylated proteins showed that the major biological functions associated with these cytoskeleton-associated proteins and phosphorylation network included cellular assembly and organization, cellular movement, cell morphology, post-translational modification, cell cycle and cell-to-cell signaling (Additional file 2: Table S1). Canonical pathways activated in HIV-infected monocytes co-cultured with HBMEC included chemokine and integrin signaling, and cell junction signaling (Additional file 3: Table S2).

**Table 2:** Phosphorylated proteins differentially expressed when normalized to total proteins or actin levels

**Phospho-protein/total protein**	**HIV vs. CONTROL**		**HIV + TAK vs. CONTROL**		**HIV vs. HIV + TAK**	
**Name**	**Fold change**	**P-value**	**Fold change**	**P-value**	**Fold change**	**P-value**
Merlin (Phospho-Ser518)	4.47	8.13E-12	1.39	8.97E-05	0.31	1.45E-10
VASP (Phospho-Ser157)	4.05	8.13E-12	1.29	0.00047	0.32	1.84E-11
ERK1-p44/42 MAP Kinase (Phospho-Tyr204)	2.38	4.31E-08	1.14	0.10	0.48	3.68E-09
Rac1/cdc42 (Phospho-Ser71)	2.24	1.28E-05	0.85	0.17	0.38	1.76E-07
Cortactin (Phospho-Tyr421)	1.20	8.13E-12	0.63	1.53E-10	0.31	1.84E-11
CaMK1-a (Phospho-Thr177)	1.93	5.75E-05	1.47	0.0092	0.76	0.00013
ERK1-p44/42 MAP Kinase (Phospho-Thr202)	1.74	6.72E-08	1.15	1.13E-05	0.66	3.71E-06
ERK3 (Phospho-Ser189)	1.68	7.74E-05	1.10	0.556	0.65	0.0035
MEK1(Phospho-Ser217)	1.47	0.00017	0.83	0.00034	0.57	4.63E-06
MKK6 (Phospho-Ser207)	0.49	8.13E-12	0.49	1.53E-10	1.01	0.96
FAK (Phospho-Tyr397)	0.44	8.13E-12	0.87	0.0552	1.97	8.0E-09
MKK7/MAP2K7 (Phospho-Ser271)	0.30	8.13E-12	0.81	0.0344	2.65	1.54E-09
**Phospho-protein/actin**	**HIV vs. CONTROL**		**HIV + TAK vs. CONTROL**		**HIV vs. HIV + TAK**	
**Name**	**Fold change**	**P-value**	**Fold change**	**P-value**	**Fold change**	**P-value**
Merlin (Phospho-Ser518)	6.24	1.95E-11	1.15	0.079	0.184	1.16E-11
VASP (Phospho-Ser157)	4.38	1.95E-11	0.88	0.088	0.20	1.16E-11
Rac1/cdc42 (Phospho-Ser71)	3.56	3.67E-08	0.83	0.14	0.23	6.98E-10
ERK1-p44/42 MAP Kinase (Phospho-Tyr204)	3.26	9.42E-10	0.88	0.13	0.27	1.16E-11
Cortactin (Phospho-Tyr421)	2.93	1.96E-11	0.73	7.19E-08	0.25	1.16E-11
ERK1-p44/42 MAP Kinase (Phospho-Thr202)	2.55	1.2E-10	0.92	0.0028	0.34	3.18E-11
MEK1(Phospho-Ser217)	2.23	2.06E-08	0.74	2.35E-06	0.33	7.81E-10
CaMK1-a (Phospho-Thr177)	1.67	0.00038	1.08	0.91	0.64	3.19E-07
ERK3 (Phospho-Ser189)	1.61	0.00011	1.06	0.91	0.65	0.0026
MKK7/MAP2K7 (Phospho-Ser271)	0.73	1.51E-06	0.87	0.23	1.18	0.038
FAK (Phospho-Tyr397)	0.61	1.96E-11	0.64	2.82E-06	1.05	0.63
MKK6 (Phospho-Ser207)	0.60	1.96E-11	0.71	2.27E-06	1.17	0.00089

P-values were adjusted using the Benjamini & Hochberg test.

Unadjusted P values, P values adjusted for multiple comparisons using Tukey post-hoc test, and P values adjusted for false discovery rate using the Benjamini & Hochberg method were all significant. Table show P values adjusted using the Benjamini & Hochberg method.

VASP: vasodilator-stimulated phosphoprotein; CaMK1: calcium/calmodulin-dependent protein kinase-1; ERK: extracellular signal-regulated kinases; MEK / MKK: mitogen-activated protein kinase kinase; FAK: focal adhesion kinase.

### Confirmation of non-productive HIV-1 infection of monocytes

It is known that HIV-1 does not productively infect human monocytes, and even for monocytes-derived macrophages, productive infection (detectable p24 or reverse transcriptase activity) is often seen from day-3 post-infection. To determine whether non-productive infection of monocytes occur, we analyzed gag and tat mRNA, and gp120 expression in freshly elutriated and HIV-exposed monocytes cultured 2 to 48 hours in media with and without human recombinant macrophage colony stimulating factor (MCSF, induce monocyte differentiation to macrophage). Quantitative real-time PCR for HIV-1 gag mRNA (Additional file 4: Figure S2A, B) showed that HIV-1 gag mRNA was present in monocytes from 2 hours post elutriation/infection, with more gag copies numbers in monocytes cultured in media without MCSF (Additional file 4: Figure S2B), compared to monocytes cultured in media containing MCSF (Additional file 4: Figure S2A). Gag mRNA copy numbers decreased over time but was still detectable in infected cells. Reverse-transcription PCR targeting tat mRNA (Additional file 4: Figure S2C, D) also showed detectable tat mRNA in both monocytes cultured in media with (Additional file 4: Figure S2C) and without (Additional file 4: Figure S2D) MCSF from 2 hours post elutriation/infection. Immunofluorescence analysis using gp120 monoclonal antibodies also showed positive staining for HIV-1 gp120 in monocytes from 2 hours post elutriation/infection (Additional file 5: Figure S3).

### Increased transcription of cortactin and Rac1, and Rac1 activation in brain tissues of HIV-infected patients

To determine whether our *in vitro* findings correlated with changes in HIV-infected humans, we analyzed brain tissues of 12 HIV-1,2-seronegative control subjects, 9 HIV-1-seropositive patients without evidence of HIVE, and 10 HIV-1-seropositive patients with HIVE and HAND. All brain tissues were from the cortex region, with 28 of the 31 samples from the frontal cortex, 2 samples from the parietal cortex, and 1 sample from the temporal cortex. Table 3 shows the age, gender, clinical history, post-mortem interval (PMI) between the time of death and autopsy, and a summary of post-mortem findings for all 31 human subjects. For seronegative controls, HIV-infected, and HIVE patients, the age ranges in years were respectively 32 to 72 (mean: 52±13.4), 27 to 54 (mean: 41.78±8.8), and 30 to 52 (mean: 40.6±7.76). For seronegative controls, HIV-infected, and HIVE patients, the PMI ranges in hours were respectively 3 to 8.5 (mean: 4.6±1.6), 2.75 to 15 (mean: 8.5±4), and 4 to 21 (mean: 9.45±5.16). No significant differences were detected in age and PMI between the seronegative, HIV-1-infected, or HIVE groups.

**Table 3:** Clinical history of brain tissues donors

**HIV-1 status**	**ID**	**Gender/Age (y)**	**PMI (h)**	**Neurocognition/Neuropathology**	**Other autopsy diagnosis**
Neg	N1	M/35	8.5	Normal/None	Mild Alzheimer gliosis
Neg	N2	N/A	N/A	Normal/None	N/A
Neg	N3	F/38	5.75	Normal/Not significant	Mild gliosis, lung and bile duct carcinoma
Neg	N4	M/32	4.25	Normal/Not significant	Cystic fibrosis, and multiorgan failure
Neg	N5	F/46	4	Normal/Not significant	Mild fibrosis, mild patchy gliosis
Neg	N6	F/49	4.5	Normal/Not significant	Hepatic cirrhosis, liver failure
Neg	N7	M/52	5.25	Normal/None	Hypertension, renal failure
Neg	N8	M/72	3	Normal/None	Hypertension, COPD
Neg	N9	M/64	3.3	Normal/None	Hepatic carcinoma
Neg	N10	M/56	3	Normal/Not significant	Lung adenocarcinoma, mild nonspecific cortical atrophy
Neg	N11	M/67	3.5	Normal/None	COPD, TB
Neg	N12	M/61	5.75	Normal/Not significant	Cardiomyopathy, mild gliosis
Pos	P1	?/46	2.75	Normal/None	N/A
Pos	P2	?/27	8	Normal/None	N/A
Pos	P3	?/37	5	Normal/None	N/A
Pos	P4	M/39	11	Normal/None	Minimal non-diagnostic abnormalities
Pos	P5	M/35	6.5	Normal/None	Non-Hodgkins lymphoma, AIDS
Pos	P6	M/54	6.5	Normal/None	AIDS
Pos	P7	M/48	15	Normal/None	Liver disease
Pos	P8	M/52	8	Normal/None	AIDS
Pos	P9	M/38	14	Normal	Pneumonia, hemophilia A, AIDS
Pos	HAD1/D1	M/30	6	HAD/HIVE	Minimal terminal anoxia
Pos	HAD2/D2	?/50	21	HAD/HIVE	N/A
Pos	HAD3/D3	?/39	12	HAD/HIVE	N/A
Pos	HAD4/D4	?/40	12	HAD/HIVE	N/A
Pos	HAD5/D5	M/47	11	HAD/HIVE	Encephalopathy, microglial nodule encephalitis, meningoencephalitis with microvascular damage
Pos	HAD6/D6	M/40	5	HAD/HIVE	Non-Hodgkin’s lymphoma, AIDS, lymphocytic meningitis
Pos	HAD7/D7	M/44	4	HAD/HIVE	Microglial nodules, astrocytosis, widespread gliosis, AIDS
Pos	HAD8/D8	M/52	5	HAD/HIVE	Atherosclerosis
Pos	HAD9/D9	M/34	11.5	HAD/HIVE	AIDS
Pos	HAD10/D10	M/38	7	HAD/HIVE	HIV encephalopathy/Leukoencephalopathy, AIDS

Neg indicates HIV seronegative; Pos, HIV seropositive; HAD, HIV-associated dementia; HIVE, HIV encephalitis; y, years; M, male; F, female; ? or N/A, not available; and PMI, postmortem interval; AIDS, acquired immunodeficiency syndrome; COPD: chronic obstructive pulmonary disease; TB, tuberculosis.

**Figure 3:**
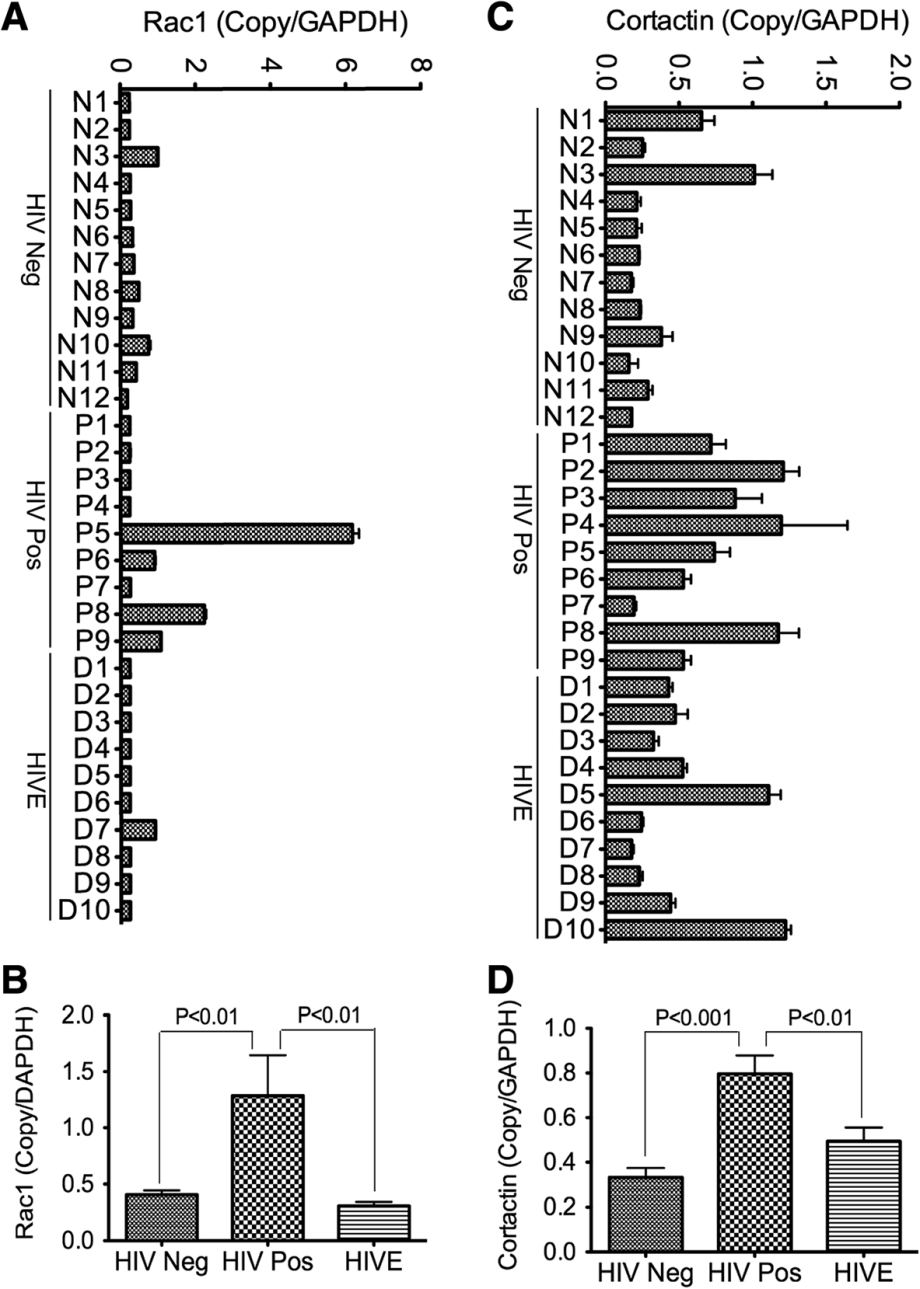
**Increased transcription of *RAC1 *and *CTTN *in brain tissues of HIV-infected humans.** Quantitative real-time PCR show overall increase in Rac1 **(A, B)** and cortactin **(C, D)** mRNA in brain tissues of HIV-positive individuals (HIV Pos, donors P1 to P9), compared to seronegative controls (HIV Neg, donors N1 to N12) and HIV-infected patients with encephalitis (HIVE, donors D1 to D10).

Quantitative real-time PCR (qRT-PCR) showed transcriptional upregulation of *RAC1* and *CTTN* in brain tissues from HIV-1-infected patients. Compared to brain tissues from seronegative and HIVE patients, brain tissues from HIV+ patients had 3-fold and 4-fold higher *RAC1* mRNA respectively (Figure 3A, B, P < 0.01), and had 2.4-fold (P < 0.001) and 1.6-fold (P < 0.01) higher *CTTN* mRNA respectively (Figure 3C, D).

**Figure 4:**
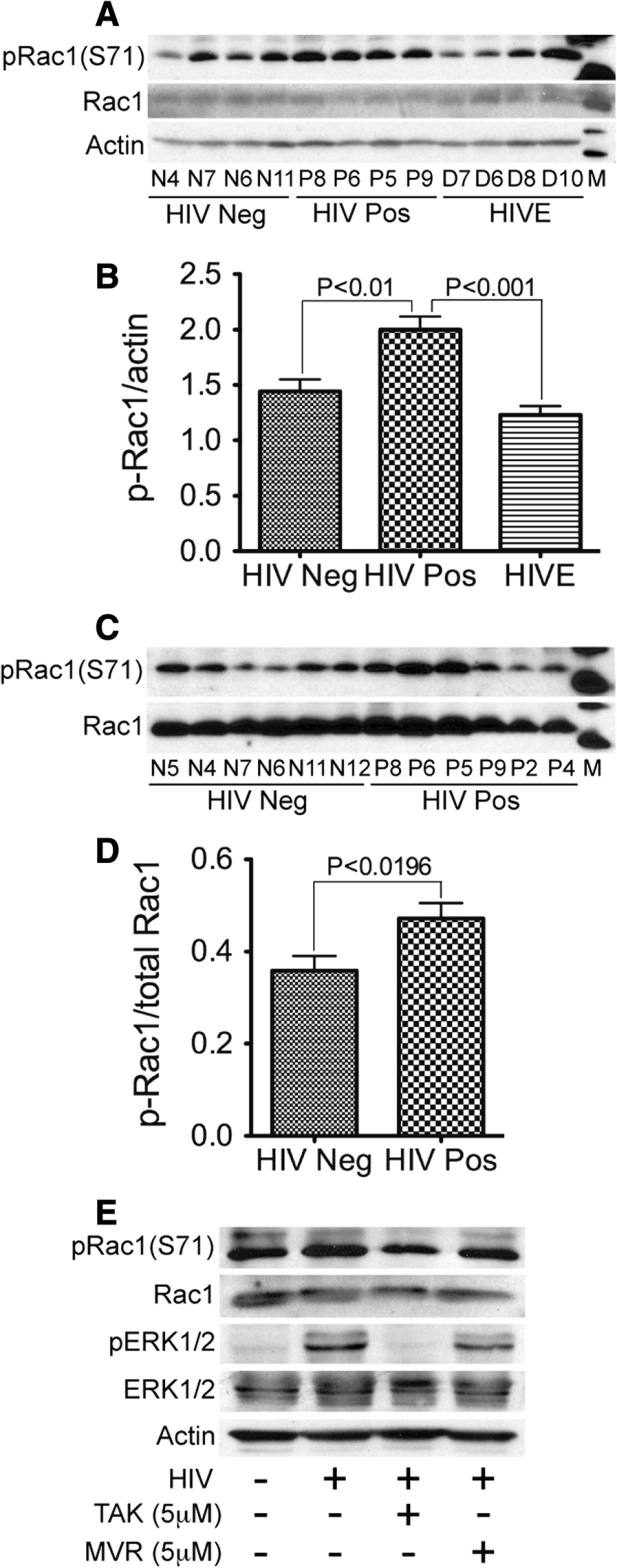
**Increased phosphorylation of Rac1(S71) in brain tissues of HIV-infected humans. (A-D)** Western blot analyses show increased levels of pRac1 (S71) in brain tissues of HIV-positive individuals (HIV Pos, donors P8, P6, P5, P9, P2, and P4), compared to HIV-infected patients with encephalitis (HIVE, donors D7, D6, D8, and D10) and seronegative controls (HIV Neg, donors N5, N4, N7, N6, N11, and N12). **(E)** Increased levels of pRac1 (S71) and pERK1 (Thr202/Tyr204) in HIV-infected monocytes co-cultured with HBMEC, compared to non-infected monocytes co-cultured with HBMEC. TAK-779 and maraviroc diminished HIV-induced phosphorylation of Rac1 and ERK1/2.

Analysis of Rac1 activation in human brain tissues showed significantly increased levels of phosphorylated Rac1(S71) in brain tissues from HIV+ patients, compared to brain tissues from seronegative controls and HIVE patients (Figure 4A-D). Additional western blot experiments confirmed our protein microarray results and showed that HIV-1 infection increased phosphorylation of Rac1 at S71, and phosphorylation of ERK1/2 in human monocytes following monocyte-endothelial communication, and the CCR5 antagonists maraviroc and TAK-779 diminished HIV-induced phosphorylation of Rac1 and ERK1/2 (Figure 4E).

**Figure 5:**
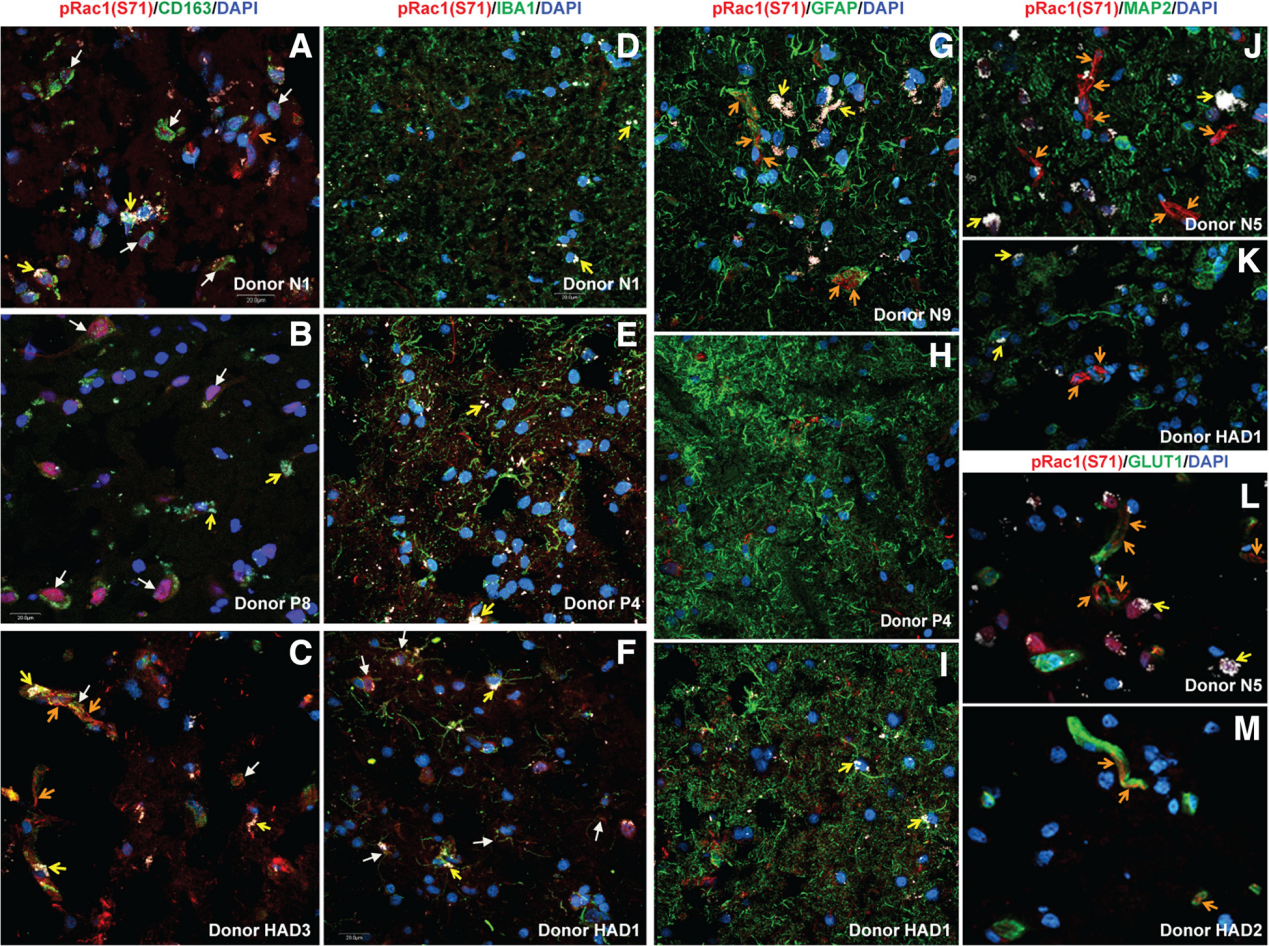
**Expression of pRac1 (S71) in human brain immune cells.** Confocal microscopy shows expression of pRac1(S71) in human brain macrophages (**A-C**, white arrows) and blood vessels tight junction strands (**A**, **C**, **G, J-M** orange arrows). Most brain tissues samples did not show significant expression of pRac1 (S71) in microglia **(D, E)**, but brain tissues of some HIVE patients showed pRac1(S71) in microglia (**F**, white arrows). Data showed no expression of pRac1 (S71) in astrocytes **(G-I)** or neurons **(J, K)**. For all experiments, a 4th laser line was used to differentiate lipofuscin-like autofluorescent pigments from antibody staining (yellow arrows). Scale bar for all panels: 20 μM.

To determine which cell type in the human brain expresses phospho-Rac1 (S71), we analyzed brain tissue sections from seronegative controls, HIV+ and HIVE patients by confocal microscopy. Data showed high expression of pRac1 (S71) in brain macrophages (Figure 5A-C, white arrows), and blood vessels, with pRac1(S71) mostly expressed on vessels tight junction strands (Figure 5A, C, G, J-M, orange arrows). Many samples did not show pRac1 expression in microglia (Figure 5D, E), but some HIVE patients showed pRac1 expression in microglia (Figure 5F, white arrows). No sample showed pRac1(S71) expression in astrocytes (Figure 5G-I) or neurons (Figure 5J, K). Because lipofuscin-like pigments can accumulate in human brain and autofluoresce over a broad excitation and emission spectra [24], we used a 4th laser line (excitation: 543-nm, emission: 595-nm) to differentiate autofluorescent pigments from antibody staining. These autofluorescent pigments are shown in white (Figure 5, yellow arrows).

## Discussion

Chemokine receptors such as CCR5 and CXCR4 play a major role in HIV-entry into target cells and viral infection [5-8]. Maraviroc, a slowly reversible CCR5 antagonist [9] and the only FDA-approved entry inhibitor, is currently used for the treatment of patients infected with M-tropic and dual-tropic HIV strains [9,25,26]. HIV infection and immune activation of MPs promotes monocyte-endothelial interactions and increased entry of MPs into the CNS [4,27-29]. In the present study, we demonstrate that HIV-1 increases expression and activation of MPs cytoskeletal-associated proteins during monocyte-endothelial interactions, and CCR5 blockers diminished these effects, diminished HIV-induced increase in monocyte adhesion to *in vitro* BBB models, and prevented viral infection.

Cytoskeletal-associated proteins activated following monocyte-endothelial communications included Rac1. To our knowledge, this is the first study to show that HIV-1 induced phosphorylation of Rac1 at S71 in MPs during monocyte-endothelial interactions, and that this is likely mediated by CCR5, since CCR5 antagonists diminished Rac1 S71 phosphorylation. This is also the first study, to our knowledge, to show that 1) HIV-1 infection in humans is associated with increased transcription of *RAC1* and *CTTN*, 2) Rac1 phosphorylation at S71 is increased in HIV+ brain tissues, and 3) cells expressing phosphorylated Rac1 (S71) in the human brain were mostly associated with brain macrophages and blood vessels. It is known that tissue damage such neuronal cell death and gliosis are common in HIVE, and this is confirmed by our immunostaining showing reduced staining for the neuronal marker MAP2 in HIVE. However, we do not believe that these tissue damages could have impacted our results on cells expressing activated Rac1, because analysis of brain tissues from HIV seronegative controls, HIV+ cases without encephalitis, and HIVE patients showed that activated Rac1 (S71) were mostly expressed in brain macrophages and blood vessels, and none of the sample analyzed showed expression of phosphorylated Rac1 (S71) in neurons or astrocytes.

Significant increases in transcription of *RAC1* and *CTTN* were observed only in brain tissues of HIV-infected individuals, compared to brain tissues of seronegative controls, or infected patients with advanced neurological complications (HIVE), suggesting that HIV-induced transcriptional regulation of *RAC1* and *CTTN* occurs early in the course of viral infection, which likely coincides with BBB breach and increased trafficking of MPs into the CNS. HIV-induced BBB dysfunction and the resulting increased entry of MPs into the CNS are well-documented to precede subsequent CNS complications such as HIVE and HAND [27-29]. Previous studies also showed increased transcriptional upregulation of proinflammatory cytokines such as IL6, and STAT1, in brain tissues of HIV+/nonencephalitic patients, compared to brain tissues of seronegative controls and HIVE patients [30], confirming that increased inflammation and inflammation-induced damages that leads to HIVE often precede the onset of HIVE. It has also been demonstrated that Rac1 activation is associated with clustering of cell adhesion molecules, increased production of reactive oxygen species, and leukocyte transendothelial migration [31,32]. In HIV-induced CNS dysfunction, such oxidative stress events and leukocyte entry into the CNS occur earlier following HIV infection, before the onset of HIVE.

Ligand binding to chemokine receptors has been shown to induce activation of signaling that regulate cellular integrins and adhesion molecules, resulting in rearrangement of the actin cytoskeleton, changes in cell morphology and migration [33]. This agrees with our current study, which shows that proteins differentially expressed and activated in HIV-infected monocytes following monocyte-endothelial communications are associated with functions such as cellular movement, cell morphology, cell-to-cell signaling, and post-translational modifications. The signaling pathways activated in HIV-infected monocytes following monocyte-endothelial interaction included those associated with integrin and cell junction signaling.

Rac1 is a small signaling G protein that, when activated, binds to other effector proteins to regulate cellular functions such as cytoskeletal reorganization, cell-cell adhesion, and motility [18], integrin- and cadherin-mediated cell-cell adhesion [19]. Rac1 is the predominant Rac isoform in monocytes, accounting for 90% of Rac expression [34]. It also has been shown that hepatitis-B virus activates Rac1, which further induces ERK1/2, AKT, and STAT phosphorylation [35]. Rac1 inactivation inhibits cell proliferation and migration [36] while Rac1 S71 phosphorylation increases filopodial structures and enhances cell motility and migration [37]. Previous studies in astroglioma cell lines also showed that Rac1 is activated during HIV-induced cell fusion and this is mediated by co-receptors and cytoskeletal actin [38], and Rac1 inhibitors decreased adhesion of U937 cells to endothelial cells [39]. Our data showed overall higher levels of Rac1 mRNA and pRac1(S71) in brain tissues from HIV-infected humans; however, the levels were not uniform and some HIV patients had more Rac1 mRNA and pRac1(S71) than others. Because the clinical information of patients used in this study did not include viral loads, we do not know whether this differential Rac1 activation and transcriptional regulation is associated with increased plasma or CSF viremia.

Ample evidence indicates that G-protein couple receptors such as CCR5 directly interact with the cytoskeleton and Rac1, and these interactions affect infectivity and migration. Binding of HIV-1 envelope glycoprotein to the CD4 receptor and CCR5 or CXCR4 co-receptors induces a signaling cascade that results in Rac1 activation and actin cytoskeletal reorganizations, changes that are required for efficient viral-mediated membrane fusion and infection [38,40]. Studies in macrophages and CCR5-transfected cells also showed that CCR5 binding to its ligands induce signaling that results in Rac1 activation, cytoskeletal reorganization and formation of lamellipodia, and these effects are blocked by a Rac dominant negative mutant [41].

Our present study confirmed the antiviral activity of maraviroc and also showed that both maraviroc and TAK-779 at 0.5-5 μM (0.25-2.57 ng/ml) inhibit HIV-1 infection of human macrophages without inducing additional cell toxicities. Studies of humans on maraviroc treatment reported a median (and range) maraviroc plasma concentrations of 94.9 (21.4–478) ng/ml [42]; 124.75 (7.3-517) ng/ml [43]; 123 (31.7–529) ng/ml [44]; and 347 (123–2678) ng/ml [45]. Maraviroc could also be quantified in the CSF of these patients, with median (and range) concentrations of 3.6 (1.83-12.2) ng/ml; [42] 2.58 (0.5–7.22) ng/ml; [43] and 102 (35–173) ng/ml [45]. The concentrations of CCR5 antagonists used in our present study were much lower than those reported in human plasma and CSF [42-45], thus indicating that our findings are relevant to *in vivo* situations in humans.

In addition to their antiviral effects, CCR5 blockers can have other beneficial effects on the immune system. Maraviroc concentrations of 0.1-4 μM lowers lipopolysaccharide-induced inflammation in adipocytes, decreasing mRNA and secretion of MCP-1, IL-8, and IL-6 [46]. Maraviroc treatment improves progressive multifocal leukoencephalopathy-immune reconstitution inflammatory syndrome [47] and improves lipid profiles in HIV patients with dyslipidemia [48]. Maraviroc also has CNS antiviral and metabolic activities that include significant reduction in plasma and CSF viral load of patients with neurological impairment [45], and increased NAA/creatinine levels [49], suggesting that maraviroc improved neuronal integrity. *Ex vivo* and *in vitro* studies showed that maraviroc significantly reduced inflammation and the chemotactic activities of peripheral blood mononuclear cells, T-lymphocytes, dendritic cells, monocytes and macrophages [50-52], and decreased lipopolysaccharide-induced MMP9 expression in astrocytes and microglia [53]. These findings showed that maraviroc could reduce inflammation and leukocyte trafficking into the CNS. Our current data showed that CCR5 blockers and CCR5 antibodies reduce HIV-induced monocyte adhesion to *in vitro* BBB models. Adhesion precedes leukocyte migration and trafficking, which suggests that CCR5 blockers and CCR5 antibodies can reduce leukocyte trafficking across the BBB. Most importantly, our data suggest that HIV infection in humans is associated with transcriptional regulation and activation of Rac1 in the CNS, and CCR5 blockers such as maraviroc could prevent Rac1 activation and improve HIV-induced CNS complications in infected humans.

## Conclusions

HIV-1 increases expression and activation of MPs cytoskeletal-associated proteins during monocyte-endothelial interactions, and CCR5 blockers diminished these effects, diminished HIV-1-induced increase in monocyte adhesion to *in vitro* BBB models, and prevented viral infection. These findings are important and have implications in cell-to-cell interactions and signaling, in HIV-1 infection and viral entry into the brain, and subsequent neurological complications. Eliminating or reducing viral reservoir is important for combatting HIV/AIDS and reducing disease burden, and the current study suggest that antiretroviral regimens containing CCR5 blockers such as maraviroc could reduce cellular trafficking across the brain endothelium, viral entry into the CNS and disease burden.

## Methods

### Monocyte isolation and HIV-1 infection

Human monocytes were obtained from HIV-1-, HIV-2-, and hepatitis B-seronegative donor leukocytes and separated by countercurrent centrifugal elutriation and infected with HIV-1_ADA_, a clade-B, M-tropic viral isolate, as previously described [54]. Briefly, freshly elutriated monocytes were re-suspended in Dulbecco’s Modified Eagles Media (DMEM) containing 2 mM L-glutamine (Invitrogen), 10% heat-inactivated human serum, 100 μg/ml gentamicin, and 10 μg/ml ciprofloxacin (Sigma); HIV-1_ADA_ was then added at MOI 0.01, and monocytes cultured overnight (for 12 hours). After the 12 hours culture, monocytes were washed 3 times with PBS to remove free viral particles, and used for co-culture experiments. MDM were obtained from monocytes by culture for 7 days in DMEM containing 2 mM L-glutamine (Invitrogen), 10% heat-inactivated human serum, 100 μg/ml gentamicin, and 10 μg/ml ciprofloxacin (Sigma) in the presence of 1,000 U/ml MCSF as previously described [54]. For experiments testing the effects of CCR5 blockers, monocytes and MDM were treated with 5 μM TAK-779 or 5 μM maraviroc for 30 min prior to viral exposure. Both TAK-779 and maraviroc were obtained from the NIH AIDS Research and Reference Reagent Program. All reagents were prescreened for endotoxin (<10 pg/ml, Associates of Cape Cod, Woods Hole, MA) and mycoplasma contamination (Gen-probe II, Gen-probe, San Diego, CA). Levels of productive viral replication were measured in culture fluids by reverse transcriptase assay [55], and toxicity of TAK-779 and maraviroc assessed by alamarBlue^™^ assay as previously described [56].

### Brain endothelial cell culture

Primary HBMEC were isolated from brain tissue obtained during surgical removal of epileptogenic cerebral cortex in adult patients as described previously [54,57]. Routine evaluation by immunostaining for von-Willebrand factor, *Ulex europaeus* lectin and CD31 demonstrated that cells were > 99% pure. Freshly isolated cells were cultured on collagen-coated 100-mm culture plates [54], and cells at passage 2 to 4 were used in this study.

### Measure of trans-endothelial electrical resistance (TEER)

For TEER measurements, HBMEC were seeded on gold-film electrode surface (Applied BioPhysics Inc., Troy, NY) and cultured to confluence. Confluent HBMEC were exposed to 5 to 20 μM of TAK-799 or maraviroc; TEER live-recorded readings were made before and after exposures. Controls consisted of non-treated HBMEC and cells treated with 0.1% saponin. To determine whether any effect of CCR5 blockers on the BBB was reversible, HBMEC were washed after 48 hours to remove TAK-779 or maraviroc, and TEER live-recorded readings were made for an additional 20 to 24 hours.

### Co-culture of monocytes with HBMEC

HIV-1-infected monocytes were added to confluent monolayers of HBMEC in 100-mm culture plates (5 monocytes for each HBMEC), and co-cultured for 2 hours. Controls consisted of mock-infected monocytes co-cultured with HBMEC, as well as infected and mock-infected monocytes not cultured with HBMEC. For experiments testing the effects of CCR5 blockers, monocytes were treated with 5 μM TAK-779 or maraviroc for 30 min before culture. Following the 2 hours co-culture, monocytes were harvested by washing HBMEC monolayer 3 times with PBS. Adherent HBMEC were checked by microscopy to ensure adequate-maximal removal of monocytes, before harvesting endothelial cells by scrapping. Each cell type was pelleted by centrifugation at 3,000-g for 10 min, followed by protein extraction.

### Protein extraction and cytoskeleton antibody microarray

Cells were lysed and protein extracted using the Protein Extraction Kit (Full Moon Biosystems, Sunnyvale, CA) according to the manufacturer’s protocol and protein concentration measured using the bicinchoninic acid assay (Pierce, Rockford, IL). The Cytoskeleton-II protein arrays and all reagents used for protein microarray analysis were from Full Moon Biosystems, except Cy3-conjugated streptavidin, which was from GE Healthcare Life Sciences (Piscataway, NJ). Each Cytoskeleton-II protein array contained 141 well-characterized phosphorylated antibodies of the cytoskeletal pathway, and corresponding total antibodies. To ensure reliability and consistency of results, each array included 6 replicates of each antibody and phospho-antibody. Additional controls on each array included 6 positive controls consisting of Cy-3 labeled antibodies, 6 negative controls containing bovine serum albumin (BSA), and empty spots containing no antibody, with the 6 replicates scattered throughout the array. The antibody microarray was performed according to the manufacturer’s protocol and slides were scanned using Full Moon Biosystems Scanning Array Service.

### Array data analysis

Each spot throughout the array was scanned to provide its signal intensity values, including positive (spots containing Cy-3 labeled antibodies) and negative (empty spots and spots containing BSA) controls. For background correction, the median value of the negative control signal was subtracted from the values of each antibody and phospho-antibody. The background corrected signal was log_2_ transformed and normalized by the mean value of beta-actin signal. For each phosphorylated antibody, the background corrected signal was normalized by the mean value of the corresponding total antibody. The fold changes between treatment groups were derived from ratios of geometric mean signal intensities. Treatment groups included: control non-infected monocytes co-cultured with HBMEC (control), HIV-infected monocytes co-cultured with HBMEC (HIV), and HIV-infected monocytes treated with CCR5 blockers and co-cultured with HBMEC (HIV + TAK). ANOVA with heterogeneous variance was used for statistical analyses of protein expression between groups, and the Tukey-Kramer method used for multiple comparisons. The Benjamini-Hochberg (BH) method was then used to control the false discovery rate. Proteins with BH adjusted p-value less than 0.05 were considered to be differentially expressed.

### Ingenuity pathway analysis

Differentially expressed and phosphorylated proteins identified in HIV-1-infected monocytes co-cultured with HBMEC, compared to non-infected monocytes co-cultured with HBMEC, or infected monocytes treated with CCR5 blockers and co-cultured with HBMEC, were analyzed using the Ingenuity Pathways Analysis 3.0 (IPA) (Ingenuity Systems). The networks obtained through IPA software describe functional relationships between proteins products based on known interactions, biological functions and canonical pathways. Using a false discovery rate of 0.05, only phospho-proteins upregulated or downregulated by at least 1.5-fold were considered; and for non-phosphorylated proteins, only those upregulated or downregulated by at least 2-fold were considered.

### Monocyte adhesion to an *in vitro* BBB model

For adhesion assays, HBMEC were plated on 96-well collagen-coated black-bottom plates and cultured to confluence. Infected monocytes (2.5 × 10^5^ cells) treated or non-treated with CCR5 blockers or CCR5 antibodies were labeled with 5-carboxyfluorescein diacetate, acetoxymethyl ester (CFDA), 10-μM/1 × 10^6^ for 1 hour, and co-cultured with HBMEC for 15 min. HBMEC were then washed 3 times with PBS, and the number of adherent monocytes was quantified by spectrophotometry (absorbance 492-nm, emission 517-nm), with a standard curve derived from a serial dilution of a known number of CFDA-labeled monocytes.

### RNA isolation and quantitative real-time PCR (qRT-PCR)

Brain tissues (cortex) from HIV-1-seropositive patients with or without neurocognitive impairment, and HIV-seronegative controls were obtained from the National NeuroAIDS Tissue Consortium and our Department of Pharmacology and Experimental Neuroscience brain bank. The clinical histories of all brain tissue donors are detailed in Table 3. Total RNA was extracted from brain tissues using the Trizol reagent (Life Technologies-Ambion, Austin, TX) according to the manufacturer’s protocol and further cleaned using Total RNA cleanup kit (Qiagen, Valencia, CA). RNA yield and quality were checked using a NanoDrop spectrophotometer (NanoDrop Technologies, Wilmington, DE) and for all samples absorbance ratio of 260/280 was ≥ 2. For each sample, cDNA was generated from 2 μg RNA using the High Capacity cDNA Reverse Transcription Kit (Applied Biosystems, Foster City, CA). A TaqMan gene detection system was used and quantification performed using the standard curve method as described in the Applied Biosystems StepOnePlus^™^ protocol. All reagents and primers were obtained from Applied Biosystems and for endogenous controls, each gene expression was normalized to GAPDH. Primer IDs were Rac1: Hs01902432_s1, cortactin: Hs01124225_m1, and GAPDH Hs99999905_m1.

### Western blot analyses

Protein extraction and Western blot analyses were performed as previously described [30,56]. ERK1/2 antibodies were from Cell Signaling (Danvers, MA), while all other antibodies were from Full Moon Biosystems. Following Western blot with phosphorylated antibodies, membranes were stripped using Restore Western Blot Stripping Buffer (Pierce) and re-blotted with the corresponding total antibody, then stripped again and re-blotted with β-actin antibody to confirm equal loading. Results were expressed as ratios of relative intensity of the phospho-protein to total protein, or β-actin.

### Immunofluorescence and confocal microscopy

Five-micrometer sections were cut from each human brain tissue, mounted on glass slides, fixed, permeabilized, and incubated 1 hour in PBS containing 3% BSA to block for non-specific binding. Tissues were incubated overnight with antibodies to phospho-Rac1(S71) and CD163, ionized calcium binding adapter molecule-1 (IBA1), glial fibrillary acidic protein (GFAP), microtubule-associated protein-2 (MAP2), or glucose transporter-1 (GLUT1) (Abcam, Cambridge, MA), followed by staining (1 hour in the dark at room temperature) with secondary antibodies coupled with Alexa Fluor-488 or −635 (Invitrogen) at 1:500 dilutions. Stained tissues were washed in PBS and mounted in Prolong Gold anti-fade reagent containing DAPI (Molecular Probes, Grand Island, NY) and examined under an Olympus FV500-IX 81 confocal laser scanning imaging system. The triple laser lines excitation were 405-nm for nucleus-stains; 484-nm for CD163, GFAP, MAP2, IBA1, or GLUT1, and 635-nm for pRac1(S71). A 4th laser line (excitation: 543-nm. Emission: 595-nm) was used to detect auto-fluorescent pigments.

### Detection of HIV-1 in infected monocytes

Freshly elutriated monocytes were re-suspended in DMEM with or without MCSF as described above, exposed to HIV-1 (MOI: 0.01) and cultured for 2 hours, 4 hours, 12 hours, 24 hours, and 48 hours. Cells were then washed three times with PBS to remove excess viral particles, and cellular RNA extracted using the Trizol reagent. Extracted RNA were further cleaned using the Qiagen RNA Cleanup kit (Qiagen, Valencia, CA). HIV-1 gag mRNA was quantified by real-time PCR as described above, using custom ordered probes and gag primers from Applied Biosystems [probe sequence: FAM-CAT CAA TGA GGA AGC TGC AGA ATG GGA TAGA-MGB; Forward (FW) primer: 5′-ACA TCA AGC CAT GCA AAT-3′ (nucleotide 1368–1388); Reverse (RV) primer: 5′-ATC TGG CCT GGT GCA ATA GG-3′ (nucleotide 1472–1453)]. All reactions were normalized to sample GAPDH (primers ID# Hs99999905_m1, Applied Biosystems).

Reverse-transcription PCR (RT-PCR) was performed using 20 ng RNA, Promega PCR master mix, and the following Tat primers: FW primer: 5′-GGA ATT CAC CAT GGA GCC AGT AGA TCC T-3′; RV primer: 5′-CGG GAT CCC TAT TCC TTC GGG CCT GT-3′ custom ordered from Integrated DNA Technology Inc. (Coralville, Iowa), in a 25 μl total reaction volume. Tat PCR reaction cycle was as followed: 94°C, 10 min initial denaturation; followed by 70 cycles of 94°C for 1 min, 60°C for 35 seconds, and final elongation at 72°C for 10 min. RT-PCR reactions were run on a 1.5% agarose gel and images captured using Syngene G-box (Syngene, Frederick, MD).

For immunofluorescence imaging of infected monocytes, 50,000 freshly elutriated monocytes were placed on poly-l-lysine coated glass coverslips, cultured for 2 to 48 hours in DMEM without MCSF in the presence of HIV-1_ADA_ (MOI: 0.01) as described above. Cells were then fixed for 20 min in cold methanol/acetone (1:1), permeabilized, and blocked for non-specific binding by incubation with 3% bovine serum albumin in PBS (10 min at 4°C). Cells were incubated with 5 μg/ml gp120 monoclonal antibody (antibody 697-30D, NIH AIDS Reagent Program) overnight at 4°C, washed 3 times with PBS, 3 to 5 minutes each wash, followed by staining (1 hour in the dark at room temperature) with secondary antibodies coupled with Alexa-488 (1:500 dilution). Stained cells were then washed with PBS 3 times, 3 to 5 minutes each wash, and mounted in Prolong Gold anti-fade reagent containing DAPI.

### Ethical approval

Removal of epileptogenic cerebral cortex tissue from adult patients, and its use for HBMEC isolation, were done after obtaining patient’s consent and ethical approval by the University of Arizona institutional review board (IRB), and primary HBMEC provided by Drs. Marlys Witte and Michael Bernas (University of Arizona, Tucson, AZ) [57]. Monocytes were obtained from adult human donor leukocytes after obtaining donor’s consent and the University of Nebraska Medical Center IRB approval. Post-mortem brain tissues obtained from brain banks did not have personal donor identifier, and did not require ethical approval for use in research.

### Statistical analyses

Data were analyzed by t-test (two-tailed) for two-group comparisons and one- or two-way ANOVA followed by Tukey’s multiple-comparisons tests using GraphPad Prism 5.0b. (GraphPad Software, La Jolla, CA). Threshold of significance level was 0.05.

## Competing interests

The authors declare that they have no competing interests.

## Authors’ contributions

SW carried out co-culture experiments, viral infection, cytotoxicity and reverse transcriptase assays, Western blot, real-time PCR, reverse transcription PCR, gel electrophoresis, immunofluorescence, cytoskeleton antibody microarray, collection of array data and ingenuity analyses, and participated in making manuscript Tables and Figures. HL carried out protein and RNA isolation from human tissues, real-time PCR and immunofluorescence. SS carried out cytoskeleton antibody microarray, cytotoxicity, TEER and adhesion assays, viral infection, and reverse transcriptase activity. FY carried out statistical analysis of protein microarray data. GDK conceived and designed the study, analyzed and interpreted data, made Figures and Tables, and wrote the manuscript. All authors read and approved the final manuscript.

## Supplementary Material

Additional file 1: Figure S1 Molecular networks of differentially expressed and phosphorylated proteins in monocytes following monocyte-endothelial communications. Interacting pathways constructed using IPA show upregulation of cytoskeletal proteins in HIV-infected monocytes following co-culture with HBMEC (A), and downregulation of these proteins in HIV-infected monocytes treated with TAK-779 and co-culture with HBMEC (B). The most significant molecular networks for differentially expressed proteins were associated with Cell-To-Cell Signaling and Interaction, Cell Death and Survival, and Developmental Disorders (A, B). Analysis of differentially phosphorylated proteins show increased phosphorylation of some proteins in HIV-infected monocytes following co-culture with HBMEC (C), and decreased phosphorylation when infected monocytes were treated with TAK-779 before co-culture with HBMEC (D). The most significant molecular networks for these differentially phosphorylated proteins were associated with Cellular Movement, Cell Cycle, Cellular Assembly and Organization (C, D). These top molecular networks identified had 11 to 15 focus proteins (proteins significantly up- or down-regulated). The intensity of node colors indicates the degree of up- (red) or down- (green) regulation. White color nodes are non-focus proteins: proteins that are biologically relevant to the pathways but were not identified as differentially expressed in our protein microarray analysis. Solid lines represent known direct interactions, dotted lines represent suspected or indirect interactions. **Abbreviations** (focus proteins are italicized): *MAP2K*: Mitogen-Activated Protein Kinase Kinase; NFAT: Nuclear Factor of Activated T-cells; IG: Immunoglobulin; AP1: Activator Protein-1; PLC: Phospholipase-C; *CAMK*: Ca2+/calmodulin-Dependent Protein Kinase; *BCAR1*(p130Cas): Breast Cancer Anti-Estrogen Resistance-1; ERK1/2: Extracellular-signal-regulated Kinase1/2; HSP90: Heat Shock Protein-90; *PRKCA*: Protein Kinase-C alpha; *RAF1*: V-Raf-1 Murine Leukemia Viral Oncogene Homolog-1; *EZR*: Ezrin; PP2A: Protein Phosphatase-2; ROCK: Rho Kinase; MLC: Myosin Light Chain; *RAC1*: Ras-related C3 botulinum toxin substrate-1; PDGF: Platelet-Derived Growth Factor; BCR: B-Cell Receptor; *CTTN*: Cortactin; *NF2*(Merlin): neurofibromin-2; PAK: p21 Activated Kinase; *PTK2*(FAK): Protein Tyrosine Kinase-2; *VASP*: Vasodilator-Stimulated Phosphoprotein; *WASF1*: WAS Protein Family, Member-1; *PLCB3*: Phospholipase C, Beta-3. Click here for file

Additional file 2: Table S1 Molecular and cellular functions associated with differentially expressed and phosphorylated proteins in HIV-infected monocytes following monocyte-endothelial interactions. Click here for file

Additional file 3: Table S2 Canonical Pathways associated with differentially expressed and phosphorylated proteins in HIV-infected monocytes following monocyte-endothelial interactions. Click here for file

Additional file 4: Figure S2 Detection of HIV-1 gag and tat mRNA in infected monocytes. Freshly elutriated monocytes were exposed to HIV-1_ADA_ (MOI: 0.01) and culture for 2 to 48 hours in media with or without MCSF as detailed in the Method section. Control consisted of non-infected monocytes (0 hour). Quantitative real-time PCR for HIV-1 gag mRNA (A, B) showed that gag mRNA was present in monocytes from 2 hour post elutriation / infection, with more gag copies numbers in monocytes cultured in media without MCSF (B), compared to monocytes cultured in media containing MCSF (A). Gag mRNA copy numbers decreased over time but was still detectable in infected cells. Reverse-transcription PCR targeting tat mRNA (C, D) also showed detectable tat mRNA in both monocytes cultured in media with (C) and without (D) MCSF from 2 hours post elutriation / infection. Click here for file

Additional file 5: Figure S3 Detection of HIV-1 gp120 in infected monocytes. Freshly elutriated monocytes were exposed to HIV-1_ADA_ and culture for 2 to 48 hours in media without MCSF as detailed in the Method section. Control consisted of non-infected monocytes (0 h). Immunofluorescence microscopy showed positive staining for HIV-1 gp120 from 2 hours post elutriation / infection. Click here for file
