# Supplementary figures and images for: HIV-1 induces cytoskeletal alterations and Rac1 activation during monocyte-blood–brain barrier interactions: modulatory role of CCR5
Source: Retrovirology. 2014 Feb 26;11:20. doi: 10.1186/1742-4690-11-20 (PMC4015682; doi:10.1186/1742-4690-11-20)

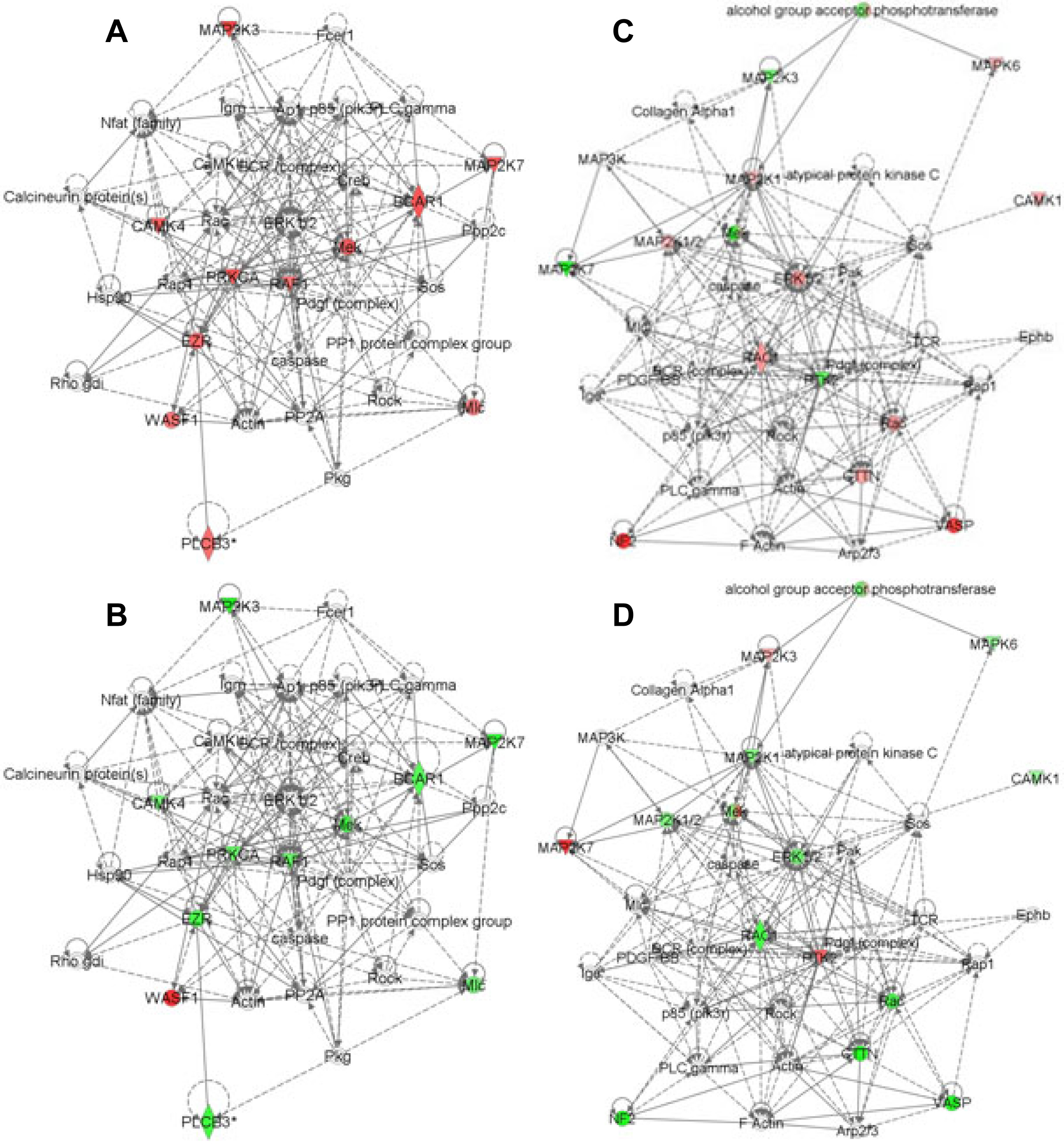

Supplement: Additional file 1: Figure S1 — Molecular networks of differentially expressed and phosphorylated proteins in monocytes following monocyte-endothelial communications. Interacting pathways constructed using IPA show upregulation of cytoskeletal proteins in HIV-infected monocytes following co-culture with HBMEC (A), and downregulation of these proteins in HIV-infected monocytes treated with TAK-779 and co-culture with HBMEC (B). The most significant molecular networks for differentially expressed proteins were associated with Cell-To-Cell Signaling and Interaction, Cell Death and Survival, and Developmental Disorders (A, B). Analysis of differentially phosphorylated proteins show increased phosphorylation of some proteins in HIV-infected monocytes following co-culture with HBMEC (C), and decreased phosphorylation when infected monocytes were treated with TAK-779 before co-culture with HBMEC (D). The most significant molecular networks for these differentially phosphorylated proteins were associated with Cellular Movement, Cell Cycle, Cellular Assembly and Organization (C, D). These top molecular networks identified had 11 to 15 focus proteins (proteins significantly up- or down-regulated). The intensity of node colors indicates the degree of up- (red) or down- (green) regulation. White color nodes are non-focus proteins: proteins that are biologically relevant to the pathways but were not identified as differentially expressed in our protein microarray analysis. Solid lines represent known direct interactions, dotted lines represent suspected or indirect interactions. Abbreviations (focus proteins are italicized): MAP2K: Mitogen-Activated Protein Kinase Kinase; NFAT: Nuclear Factor of Activated T-cells; IG: Immunoglobulin; AP1: Activator Protein-1; PLC: Phospholipase-C; CAMK: Ca2+/calmodulin-Dependent Protein Kinase; BCAR1(p130Cas): Breast Cancer Anti-Estrogen Resistance-1; ERK1/2: Extracellular-signal-regulated Kinase1/2; HSP90: Heat Shock Protein-90; PRKCA: Protein Kinase-C alpha; RAF1: V-Raf-1 Mu [file 1742-4690-11-20-S1.jpg]

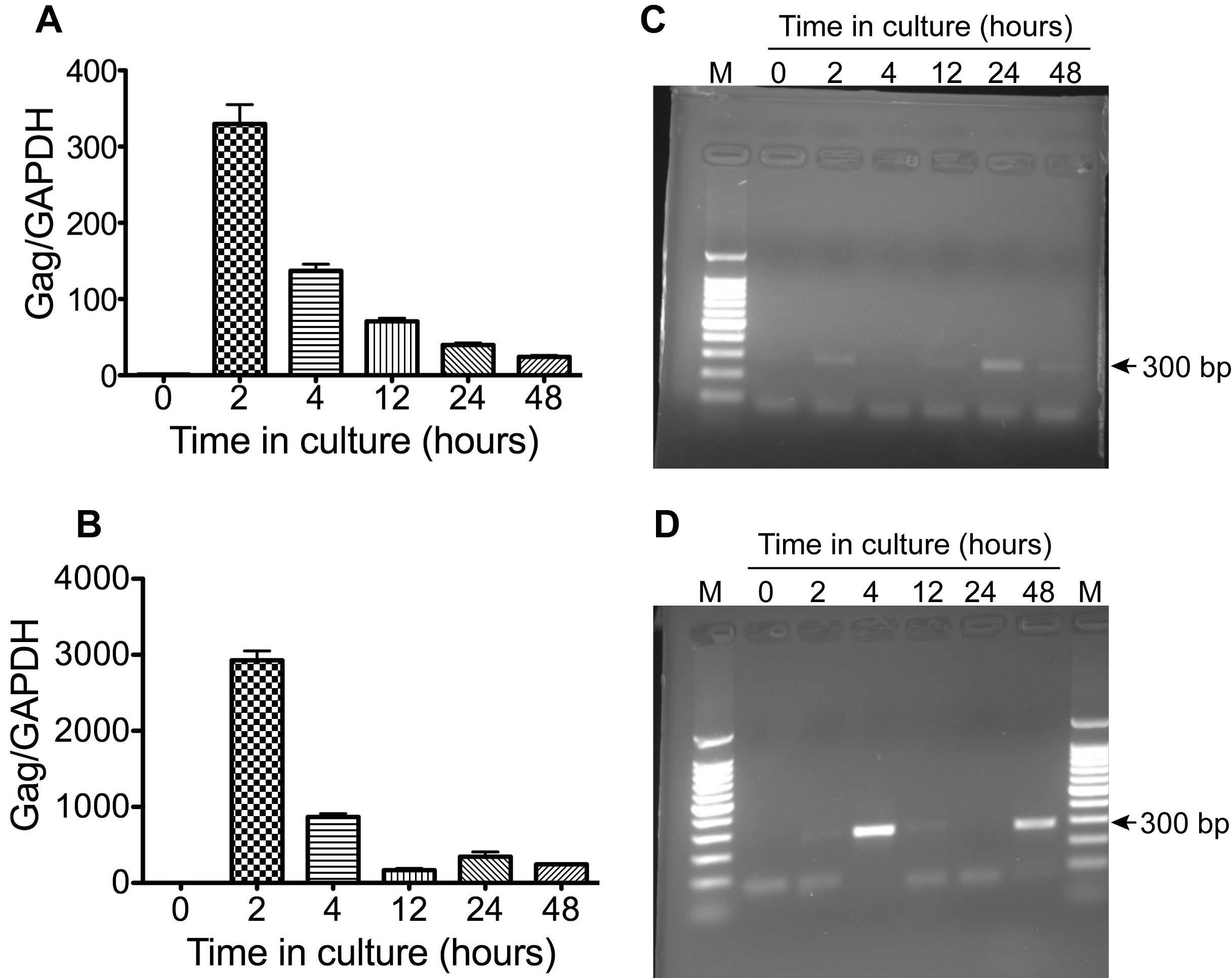

Supplement: Additional file 4: Figure S2 — Detection of HIV-1 gag and tat mRNA in infected monocytes. Freshly elutriated monocytes were exposed to HIV-1ADA (MOI: 0.01) and culture for 2 to 48 hours in media with or without MCSF as detailed in the Method section. Control consisted of non-infected monocytes (0 hour). Quantitative real-time PCR for HIV-1 gag mRNA (A, B) showed that gag mRNA was present in monocytes from 2 hour post elutriation / infection, with more gag copies numbers in monocytes cultured in media without MCSF (B), compared to monocytes cultured in media containing MCSF (A). Gag mRNA copy numbers decreased over time but was still detectable in infected cells. Reverse-transcription PCR targeting tat mRNA (C, D) also showed detectable tat mRNA in both monocytes cultured in media with (C) and without (D) MCSF from 2 hours post elutriation / infection. [file 1742-4690-11-20-S4.jpg]

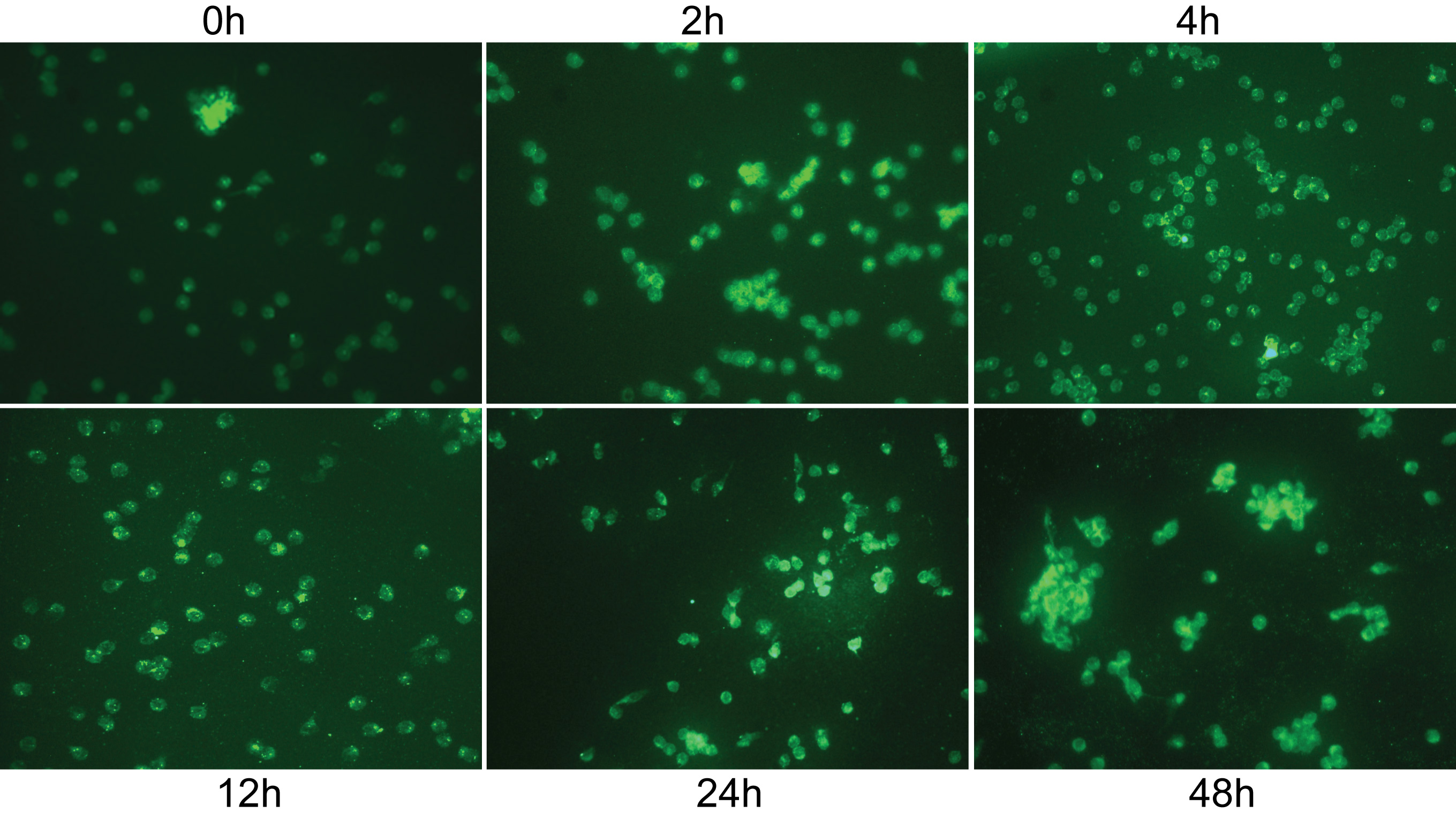

Supplement: Additional file 5: Figure S3 — Detection of HIV-1 gp120 in infected monocytes. Freshly elutriated monocytes were exposed to HIV-1ADA and culture for 2 to 48 hours in media without MCSF as detailed in the Method section. Control consisted of non-infected monocytes (0 h). Immunofluorescence microscopy showed positive staining for HIV-1 gp120 from 2 hours post elutriation / infection. [file 1742-4690-11-20-S5.jpg]
