# Supplementary material for: HIV-1 induces cytoskeletal alterations and Rac1 activation during monocyte-blood–brain barrier interactions: modulatory role of CCR5
Source: Retrovirology. 2014 Feb 26;11:20. doi: 10.1186/1742-4690-11-20 (PMC4015682; doi:10.1186/1742-4690-11-20)
Supplement: Additional file 2: Table S1 — Molecular and cellular functions associated with differentially expressed and phosphorylated proteins in HIV-infected monocytes following monocyte-endothelial interactions. [file 1742-4690-11-20-S2.doc]

**Additional file 2: Table S1.** **Molecular and cellular functions associated with differentially expressed and phosphorylated proteins in HIV-infected monocytes following monocyte-endothelial interactions.**

| **Differentially expressed total proteins** | | |
| --- | --- | --- |
| ***Molecular and cellular functions*** | **P-value** | **N** |
| Cellular Assembly and Organization | 9.10E-08 - 9.06E-03 | 11 |
| Cellular Movement | 1.75E-07 - 9.06E-03 | 11 |
| Cell Morphology | 2.35E-07 - 9.76E-03 | 11 |
| Cellular Function and Maintenance | 3.24E-07 - 9.06E-03 | 11 |
| Cellular Development | 3.74E-07 - 9.68E-03 | 9 |
| **Differentially expressed phospho-proteins** | | |
| ***Molecular and cellular functions*** | **P-value** | **N** |
| Cell Cycle | 4.42E-11 - 5.12E-03 | 10 |
| Cell Signaling | 6.64E-10 - 5.07E-03 | 9 |
| Cellular Movement | 1.23E-09 - 5.24E-03 | 8 |
| Cell Morphology | 3.71E-09 - 5.12E-03 | 10 |
| Post-translational Modification | 4.87E-09 - 5.07E-03 | 10 |
| Differentially expressed total proteins were normalized to beta actin levels; differentially expressed phospho-proteins were normalized to total protein levels.  N: number of associated proteins | | |
