# Supplementary material for: HIV-1 induces cytoskeletal alterations and Rac1 activation during monocyte-blood–brain barrier interactions: modulatory role of CCR5
Source: Retrovirology. 2014 Feb 26;11:20. doi: 10.1186/1742-4690-11-20 (PMC4015682; doi:10.1186/1742-4690-11-20)
Supplement: Additional file 3: Table S2 — Canonical Pathways associated with differentially expressed and phosphorylated proteins in HIV-infected monocytes following monocyte-endothelial interactions. [file 1742-4690-11-20-S3.doc]

**Additional file 3: Table S2. Canonical Pathways associated with differentially expressed and phosphorylated proteins in HIV-infected monocytes following monocyte-endothelial interactions.**

| **Differentially expressed total proteins** | | |  |
| --- | --- | --- | --- |
| **Canonical pathways** | **P-value** | | **N** |
| Chemokine Signaling | 3.08E-12 | | 6 |
| Melatonin Signaling | 3.37E-12 | | 6 |
| Role of NFAT in Cardiac Hypertrophy | 1.02E-11 | | 7 |
| Cholecystokinin/Gastrin-mediated Signaling | 3.23E-11 | | 6 |
| GNRH Signaling | 1.52E-10 | | 6 |
| **Differentially expressed phospho-proteins** | | |  |
| **Canonical pathways** | | **P-value** | **N** |
| GNRH Signaling | | 7.51E-11 | 6 |
| Germ Cell-Sertoli Cell Junction Signaling | | 2.29E-10 | 6 |
| Integrin Signaling | | 9.39E-10 | 6 |
| HGMB1 Signaling | | 1.9E-09 | 5 |
| HGF Signaling | | 2.74E-09 | 5 |

Differentially expressed total proteins were normalized to beta actin levels; differentially expressed phospho-proteins were normalized to total protein levels.

NFAT: nuclear factor of activated T-cells; GNRH: gonadotropin-releasing hormone; HGMB1: High-mobility group box 1; HGF: Hepatocyte growth factor; N: number of associated proteins.
